# Additional Thirty Seconds Observation with Linked Color Imaging Improves Detection of Missed Polyps in the Right-Sided Colon

**DOI:** 10.1155/2018/5059834

**Published:** 2018-07-08

**Authors:** Naohisa Yoshida, Yutaka Inada, Ritsu Yasuda, Takaaki Murakami, Ryohei Hirose, Ken Inoue, Osamu Dohi, Yuji Naito, Kiyoshi Ogiso, Yukiko Morinaga, Mitsuo Kishimoto, Eiichi Konishi, Yoshito Itoh

**Affiliations:** ^1^Department of Molecular Gastroenterology and Hepatology, Kyoto Prefectural University of Medicine, Graduate School of Medical Science, Kyoto, Japan; ^2^Department of Gastroenterology, Fukuchiyama City Hospital, Kyoto, Japan; ^3^Department of Gastroenterology, Osaka General Hospital of West Japan Railway Company, Osaka, Japan; ^4^Department of Surgical Pathology, Kyoto Prefectural University of Medicine, Graduate School of Medical Science, Kyoto, Japan

## Abstract

**Background and Aims:**

Missed polyps are a pitfall of colonoscopy. In this study, we analyzed the efficacy of an additional 30 seconds observation using linked color imaging (LCI) for detecting adenoma and sessile serrated adenoma/polyp (SSA/P).

**Materials and Methods:**

We enrolled patients undergoing colonoscopy from February to October 2017 in two institutions. In all patients, the cecum and ascending colon were observed with white light imaging (WLI) first. The colonoscope was inserted again, and the cecum and ascending colon were observed for an additional 30 seconds using either LCI or WLI. The method for the 30 sec observation was to insufflate the cecum and ascending colon sufficiently and observe them in a distant view, because the length of the second observation was determined to be precisely 30 sec. For the second observation, LCI was performed for the first 65 patients and WLI for the next 65. Adenoma and SSA/P detection rate (ASDR) in the second observation were examined in both groups. According to a pilot study, the sample size was estimated 65.

**Results:**

In the first observation, ASDR were 30.7% in the LCI group and 32.2% in the WLI group (*p* = 0.85). For the second observation, 13 polyps were detected in the LCI group and 5 polyps in the WLI group (*p* = 0.04). Additionally, ASDR for the second observation were 18.5% and 6.1%, respectively (*p* = 0.03). There were no significant differences between the LCI and WLI groups with respect to morphology (ratio of polypoid) (38.5% versus 60.0%, *p* = 0.52) and histology (ratio of adenoma) (92.3% versus 100.0%, *p* = 0.91). Total adenoma and SSA/P number were 48 in the LCI group and 36 in the WLI group (*p* = 0.02).

**Conclusion:**

The 30 seconds additional observation with LCI improved the detection of adenoma and SSA/P in the right-sided colon.

## 1. Introduction

Removal of adenomatous polyps by colonoscopy has been proven to prevent colorectal cancer and associated with the reduction in the incidence of proximal and distal colorectal cancers [1–3]. Colonoscopy is the most effective tool for detecting colorectal adenomas. However, the rate of missed polyps by white light imaging (WLI) observation was reported at 20–27% [4, 5]. There are some risk factors for missed polyps, such as flat morphology, smaller size, presence in the ascending colon, male sex, patients with multiple polyps at their first colonoscopy, and patients with a history of polyps [6, 7].

A LASER endoscopic system (LASEREO: Fujifilm Co., Tokyo, Japan) was developed in 2012. It allows blue laser imaging (BLI), BLI-bright, and linked color imaging (LCI) as narrow band light observation [8–11]. BLI is used with magnification and is useful for diagnosing various gastrointestinal cancers. BLI-bright is brighter than BLI and is expected to improve tumor detection. For colorectal tumors, BLI-bright makes a neoplastic lesion appear brownish in color and makes it easier to detect. Previously, we reported that BLI-bright improved numbers of polyps and adenomas more significantly than WLI did in a multicenter study [12]. However, there are several limitations in the use of BLI and BLI-bright. One of the limitations is that the residual liquid becomes reddish, which worsens the endoscopic view. Narrow band imaging (NBI) also has this limitation. The other limitation is that the endoscopic view using BLI and BLI-bright is darker than in WLI. However, LCI is brighter than BLI and BLI-bright and differentiates a lesion from the surrounding mucosa by making it appear reddish and the surrounding mucosa appears whitish, making the lesion easy to visualize. Moreover, residual liquid becomes yellowish with LCI. We previously reported that LCI improved polyp visibility using endoscopic figures and movies [13, 14]. However, there are no previous reports about whether LCI can decrease missed polyps.

In this study, we developed an additional 30 seconds (sec) observation as a second observation with LCI for decreasing missed adenoma and sessile serrated adenoma and polyp (SSA/P) and analyzed the efficacy of this method.

## 2. Materials and Methods

**Figure 1:**
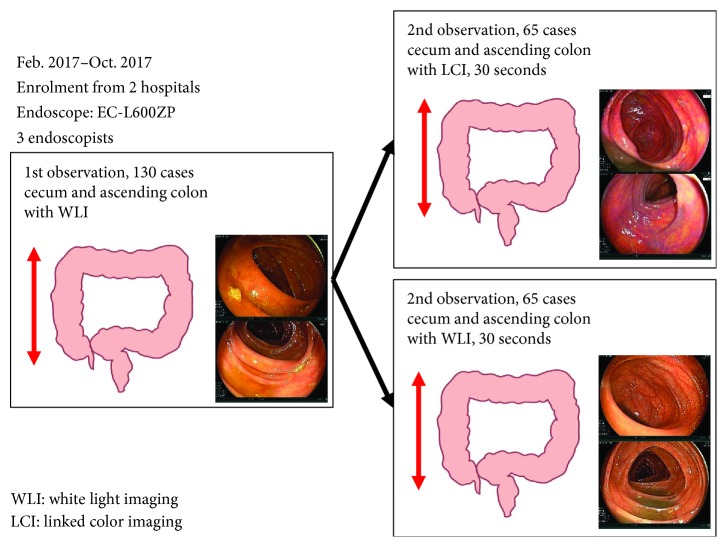
Study flow of the first observation and the second observation.

This was an observational study and was conducted at two affiliated hospitals: the Department of Molecular Gastroenterology and Hepatology, Kyoto Prefectural University of Medicine, and the Department of Gastroenterology, North Medical Center, Kyoto Prefectural University of Medicine. We examined 130 consecutive patients who received total colonoscopy using LCI and WLI with the LASER endoscopic system (LL-4450 light source, the VP-4450HD, Fujifilm Co., Tokyo, Japan) and the LASER endoscope (EC-L600ZP) from February 2017 to October 2017. We divided all patients into a LCI or a WLI group. First, in all patients, the cecum and ascending colon were observed with WLI as the first observation (Figure 1). Next, we inserted the colonoscope in the cecum again, and the cecum and ascending colon were observed for an additional 30 sec using either LCI or WLI as the second observation. In the first 65 cases, LCI was performed, and in the next 65 cases, WLI was used. The method for the 30 sec observation was to insufflate the cecum and the ascending colon sufficiently and observe them not in a close view, but in a distant view, because the length of the second observation was determined to be precisely 30 sec. Thus, even if the observation of the cecum and ascending colon had not finished during the 30 sec, the second observation would have finished. In this study, the number of adenoma and SSA/P detected in the first observation and the second observation was examined. The diagnosis of adenoma and SSA/P was performed with BLI magnification according to previous reports and local protocols based on the Preservation and Incorporation of Valuable Endoscopic Innovations (PIVI) statements, and all polyps diagnosed as either adenoma or SSA/P were resected using polypectomy, endoscopic mucosal resection (EMR), and endoscopic submucosal dissection (ESD) according to size and morphology [9, 15, 16]. Polyps diagnosed as hyperplastic polyps and inflammatory polyps were diagnosed with biopsy.

As the main purpose of this study, the number of adenoma and SSA/P in the second observation was analyzed in the LCI group compared to that in the WLI group. The number of adenoma and SSA/P in the first observation was also analyzed both in the LCI and WLI groups, respectively. Moreover, adenoma detection rates (ADR) and adenoma and SSA/P detection rates (ASDR) both in the first and second observations were calculated. Polyp characteristics such as size, location, morphology, and histology were examined in each group. Additionally, patient characteristics such as mean age, sex, insertion time, first observation time, local Boston bowel preparation score (BBPS) for the cecum and ascending colon, usage of antispasm drugs, and sedation were examined. Overall, adenoma and SSS/P numbers, adenoma numbers, ASDR, and ADR in the first and second observations in each group were analyzed. Removal time for detected polyps was not included for either the 1st observation or the 2nd observation. Moreover, polyps detected during removal were not included in this study.

The inclusion criteria were as follows: patients receiving total colonoscopies for follow-up of polyps, surveillance after polyp or cancer resection, and positive fecal occult blood performed by three experts (N.Y., Y.I., and T.M.). We excluded patients with recurrent lesions after a previous EMR and polypectomy, and T1–T4 colorectal cancers. We also excluded patients who underwent surgical operations of the cecum or the ascending colon. Of the three endoscopists, all had performed more than 1000 colonoscopies and 50 withdrawing colonoscopies with LCI prior to this study. The size of a polyp was defined by its maximum diameter and was calculated in accordance with the size of the snares and biopsy forceps. Polyps were divided into polypoid and nonpolypoid types, according to the Paris classification [17]. Histological diagnosis was performed according to the World Health Organization classification, and intramucosal cancer was categorized as adenoma in this study [18].

Regarding bowel preparation, patients followed a low-residue diet and took 10 mL of sodium picosulfate with 200 mL of water one day before the examination. All patients also received a total of 1.0 L of a highly concentrated polyethylene glycol solution with ascorbic acid (MOVIPREP; Ajinomoto Pharma Co., Ltd., Tokyo, Japan) and more than 0.5 L of water in the morning on the day of the examination according to our previous report [19].

All patients provided written informed consent to participate in this study. This study was conducted in accordance with the World Medical Association Helsinki Declaration. This study was also approved by the institutional review board and the ethics committees of Kyoto Prefectural University of Medicine.

### 2.1. Statistical Assessment

Regarding the sample size, we hypothesized that LCI improves the number of adenoma and SSAP by 20% in the second observation according to our pilot study of 20 cases by a single expert endoscopist (N.Y.). Using the sign test, the *α* error was calculated at 5% and the *β* error at 20%. The calculated minimum sample size was 47. We considered the exclusion cases and finally determined the sample size to be 65. The Mann–Whitney *U* test, Wilcoxon signed-rank test with Bonferroni correction, and chi-squared test (SPSS version 22.0 for Windows, IBM Japan, Ltd., Tokyo, Japan) were used in this study. Continuous variables such as patient age and tumor size were analyzed using the Mann–Whitney *U* test. A *p* value of < 0.05 was considered statistically significant.

## 3. Results

The patients' characteristics in the LCI group and the WLI group are shown in Table 1. The first observation times (sec) for each group were 162 ± 80 in the LCI group and 169 ± 112 in the WLI group (*p* = 0.77). There was no significant difference in the ratio of local BBPS ≥ 2 for the cecum and the ascending colon (LCI versus WLI: 84.6 versus 87.6, *p* = 0.61). On the contrary, regarding poor preparation cases showing BBPS 0 or 1, there were 10 in the LCI group and 8 in the WLI group.

The results of the first observation using WLI in the LCI and WLI groups are shown in Table 2. The numbers of adenoma and SSA/P in the LCI group and the WLI group were 35 and 31, respectively (*p* = 0.29). Regarding polyp location, the ratios of cecal location were 14.3% in the LCI group and 29.0% in the WLI group (*p* = 0.14). The ratios of polypoid morphology were 48.6% in the LCI group and 64.5% in the WLI group (*p* = 0.19). Histology showed that the ratios of adenomas were 88.6% in the LCI group and 80.6% in the WLI group (*p* = 0.37). ASDRs of the LCI and WLI groups were 30.7% and 32.3%, respectively (*p* = 0.85), and ADRs of the LCI and WLI groups were 26.2% and 26.2%, respectively (*p* = 1.0).

**Figure 2:**
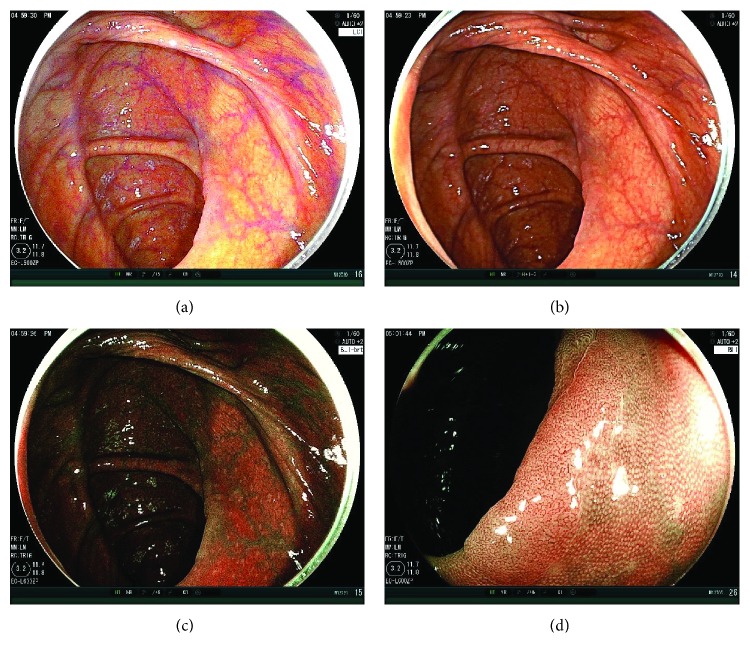
Case presentation. (a) A nonpolypoid adenomatous polyp 10 mm in size on the ascending colon. The first WLI observation could not detect this tumor. LCI could detect the tumor in a distant view. The endoscopic view was bright, and the tumor was on the fold. It was well-visualized as a little pinkish lesion under LCI compared to the surrounding mucosa. (b) WLI image of this tumor was taken afterwards. The color of it was almost similar to the surrounding tumor. (c) The BLI-bright image of it was also taken. The endoscopic view was a little dark, but the color of the tumor became brownish. (d) BLI magnification showed an adenomatous vessel pattern and surface pattern.

**Figure 3:**
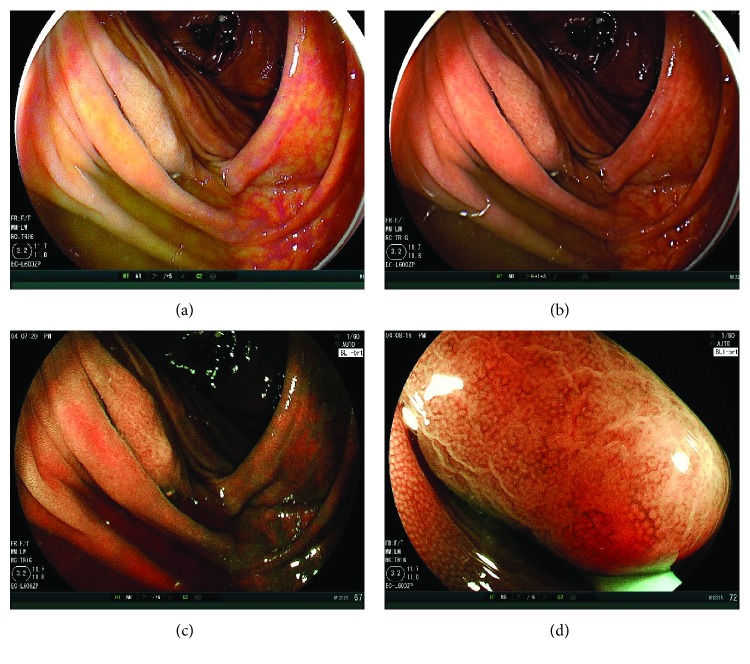
Case presentation. (a) A nonpolypoid polyp (SSA/P) 15 mm in size on the ascending colon under poor preparation. The first WLI observation could not detect this tumor. There were two other polyps which the first observation with WLI could detect in this patient. LCI could detect the tumor in a distant view. The endoscopic view was bright, and the tumor was on the fold. The color of it was more whitish under LCI compared to the surrounding mucosa. Residual liquid became still yellowish in LCI. (b) WLI image of this tumor was taken afterwards. The color of it was almost similar to the surrounding tumor. (c) The BLI-bright image of it was also taken. The endoscopic view was a little dark, and the residual liquid became reddish. The color of the tumor became brownish. (d) BLI magnification showed a dilated vessel pattern and a dilated surface pattern, consistent with typical SSA/P findings.

The results of the 30 sec second observation using LCI or WLI are shown in Table 3. The numbers of adenoma and SSA/P in the LCI and WLI groups were 13 and 5, respectively (*p* = 0.04) (Figures 2 and 3). Polyp numbers in cases with BBPS 0 or 1 were 1 in the LCI group and 1 in the WLI group (*p* = 0.92). Regarding the comparison between LCI and WLI, there were no differences between polyp sizes (mm) (5.6 ± 4.1 versus 2.2 ± 0.4, *p* = 0.33), the ratio of cecal location (23.1% versus 40.0%, *p* = 0.37), the ratio of polypoid morphology (38.5% versus 60.0%, *p* = 0.52), and the ratio of adenoma histology (92.3% versus 100.0%, *p* = 0.91). ASDRs of LCI and WLI were 18.5% and 6.1%, respectively (*p* = 0.03), and ADRs of LCI and WLI were 16.9% and 6.1%, respectively (*p* = 0.05).

The overall results of the first and second observations are shown in Table 4. The overall adenoma and SSA/P numbers in the first and second observations were 48 in the LCI group and 36 in the WLI group, respectively (*p* = 0.02). Additionally, the overall adenoma numbers of the first and second observations were 43 in the LCI group and 30 in the WLI group, respectively (*p* = 0.02). The overall ASDRs of the LCI and WLI groups were 43.1 and 35.4, respectively (*p* = 0.36), and the overall ADRs of the LCI and WLI groups were 36.9 and 30.8 (*p* = 0.45), respectively. Regarding the LCI group, the numbers of adenoma and SSA/P in the first and second observations were significantly higher than in the first observation (48 versus 35, *p* = 0.017) though there was no significant difference about that in the WLI group (36 versus 31, *p* = 0.38). The second observation with LCI also increased ASDR from 30.7% to 43.1% and increased ADR from 26.2% to 36.9%, but there were no significant differences between the first observation and the first + second observations (*p* = 0.14, *p* = 0.18).

## 4. Discussion

In our study, a 30 sec additional observation with LCI could improve overall adenoma and SSA/P numbers and overall adenoma numbers significantly more than those detected with WLI. Additionally, it significantly increased the adenoma and SSA/P numbers than the numbers in the first observation.

Our previous randomized controlled multicenter study conducted at 8 Japanese academic institutions showed that BLI-bright (489 cases) improved the mean number of adenomatous polyps detected per patient in comparison to WLI (474 cases) (1.27 ± 1.73 versus 1.01 ± 1.36, *p* = 0.008) and also improved the mean number of total polyps per patient in comparison to WLI (1.84 ± 2.09 versus 1.43 ± 1.64, *p* = 0.001) [11]. However, an observation using BLI-bright is not widely accepted. One of the reasons is that BLI-bright required longer observation times than WLI did (9.5 ± 3.8 min versus 8.4 ± 2.9 min, *p* < 0.001). The other reason is that BLI-bright is less bright than WLI and the reddish residual liquid in BLI-bright may become a disadvantage. Generally, poor preparation is detected in 20–25% of all colonoscopies [20, 21]. In those cases, BLI-bright is no more useful than WLI. As our case presentation showed, residual liquid disturbed the observation in BLI-bright. Additionally, NBI is not useful in poor preparations due to the same reasons, although current NBI systems and endoscopes showed positive results for polyp detection compared to the previous ones [22–24]. However, residual liquid is not reddish in LCI compared to BLI-bright. Thus, we suspect that LCI may be more useful for polyp detection than BLI-bright. In fact, a multicenter study from China proved that LCI increased ADR (LCI versus WLI: 37% versus 28%; 95% confidence interval, 2.3%–19.4%) [25]. The study also revealed that LCI significantly improved the rate of adenoma and SSA/P detected, more than WLI did (91% versus 73%, *p* < 0.001).

Our previous study showed that LCI improved polyp visibility more than WLI and BLI-bright did [13]. In brief, we used our original polyp visibility score (score 1: poor visibility, score 4: excellent visibility) and evaluated recorded short movies using LCI, BLI-bright, and WLI in 101 colorectal polyps. The mean polyp visibility scores of LCI were higher than those of WLI (2.86 ± 1.08 versus 2.54 ± 1.15, *p* < 0.001) and were also higher than those of BLI-bright (2.86 ± 1.08 versus 2.73 ± 1.47, *p* < 0.001). Additionally, LCI decreased the number of polyps detected showing poorer polyp visibility scores (score 1 or 2) than WLI did (experts: 35.6% versus 49.6%, *p* < 0.015; nonexperts: 33.6% versus 50.5%, *p* = 0.046). A similar study using polyp visibility scores showed that LCI improved the endoscopic visualization of nongranular colorectal lesions [26]. It also showed the efficacy of LCI for SSA/P. Moreover, our previous study showed the efficacies of LCI for nonpolypoid tumors and SSA/P [13]. Another study of ours showed the efficacy of LCI for diminutive polyps less than 5 mm in size [14]. In the study, color difference values between the tumor and the surrounding mucosa were calculated among endoscopic figures of 54 colorectal polyps taken by WLI and LCI for evaluating polyp visibility. Color difference value is thought as an objective indicator for polyp visibility. The study showed that LCI improved color difference values more than WLI did (33.6 ± 13.9 versus 20.7 ± 13.6, *p* < 0.001).

LCI presents the problem of halation in the endoscopic view due to its powerful brightness, which could lead to missed polyps, although it did not affect the results in the current study. The multicenter crossover study using LCI and WLI from China showed that there were 12 adenomas which were not detected in LCI, but were detected in WLI among 150 total adenomas [25]. Additionally, there were 29 polyps which were not detected in LCI, but were detected in WLI among 308 total polyps. We suggested that these outcomes were partially due to the powerful brightness of LCI. Missed polyps due to LCI should be examined in detail. However, the additional 30-second 2nd observation proposed in our study did not confer any additional risk of missed polyps due to LCI, because LCI is only used for second observations. In spite of this short 2nd observation time with LCI, it significantly increased the adenoma and SSA/P numbers than the numbers in the first observation. It also increased ASDR from 30.7% to 43.1% and increased ADR from 26.2% to 36.9% though it did not show significant difference (*p* = 0.14, *p* = 0.18).

Regarding serrated polyps, there are three types of polyps such as hyperplastic polyp, traditional serrated adenoma (TSA), and SSA/P [27]. TSA and SSA/P have an increased risk of progression to colorectal cancer [28, 29]. Thus, we analyzed not hyperplastic polyps but SSA/Ps in this study. Because of that, we could not calculate the polyp detection rate including hyperplastic polyps though most of previous similar studies did so.

There were some limitations in our study. This study was a parallel design without formal randomization, and a small number of patients were enrolled in two hospitals though the sample number was calculated statistically. Only three endoscopists performed all colonoscopies. There was a slight difference in the sedation rate between the WLI group and the LCI group though it was not significant. It might affect polyp detection. Though our study showed significant improvement in LCI about polyp detection in the study setting, more studies are needed to show whether this improvement is achieved in real-life settings.

## 5. Conclusions

Our study showed that a 30 sec additional observation with LCI significantly improved the number of adenoma and SSA/P in comparison to WLI. We think that our method will be easily accepted and is a less risky way for improving missed polyps.

## Figures and Tables

**Table 1:** Clinical characteristics in the LCI and WLI groups.

	LCI	WLI	*p* value
Case number	65	65	
Age, years, mean ± SD	63.5 ± 13.0	68.1 ± 14.3	0.10
Sex, %, *n* men : women	60.0 : 40.0 34 : 31	62.3 : 37.7 38 : 27	0.48
Insertion time, sec	284 ± 151	328 ± 202	0.33
First WLII observation time, sec	162 ± 80	169 ± 112	0.77
BBPS ≥ 2, %, *n*	84.6, 55	87.6, 57	0.61
Antispasm drug, %, *n*	80.0, 57	71.5, 58	0.78
Sedation, %, *n*	23.1, 15	12.3, 8	0.11

LCI: linked color imaging; WLI: white light; BBPS: Boston Bowel Preparation Score; SD: standard deviation.

**Table 2:** Results of first observation in the LCI and WLI groups.

	LCI 65 cases	WLI 65 cases	*p* value
First observation Ad and SSA/P number, *n*	35	31	0.29
Polyp size, mm, mean ± SD (range)	4.8 ± 3.6 (2–20)	4.0 ± 2.8 (2–15)	0.30
Location, %, *n* C : A	14.3 : 85.7 5 : 30	29.0 : 71.0 9 : 22	0.14
Morphology, %, *n* Polypoid: nonpolypoid	48.6 : 51.4 17 : 18	64.5 : 35.5 20 : 11	0.19
Histology Ad : SSA/P	88.6 : 11.4 31 : 4	80.6 : 19.4 25 : 6	0.37
First observation ASDR, %, *n*	30.7 20	32.3 21	0.85
First observation ADR, %, *n*	26.2 17	26.2 17	1.00

LCI: linked color imaging; WLI: white light; Ad: adenoma; SSA/P: sessile serrated adenoma and polyp; C: cecum; A: ascending colon; ASDR: adenoma and SSA/P detection rate; ADR: adenoma detection rate; SD: standard deviation.

**Table 3:** Results of the second observation in the LCI and WLI groups.

	LCI 65 cases	WLI 65 cases	*p* value
Second observation Ad and SSA/P number, *n*	13	5	0.04
Polyp number in cases with BBPS 0 or 1 in first observation	1	1	0.92
Polyp size, mm, mean ± SD (range)	5.6 ± 4.1 (2.0–15.0)	2.2 ± 0.4 (2.0–3.0)	0.33
Location, %, *n* C : A	23.1 : 76.9 3 : 10	40.0 : 60.0 2 : 3	0.37
Morphology, %, *n* Polypoid : nonpolypoid	38.5 : 61.5 5 : 8	60.0 : 40.0 3 : 2	0.52
Histology Ad : SSA/P	92.3 : 7.7 12 : 1	100.0 : 0 5 : 0	0.91
Second observation ASDR, %, *n*	18.5 12	6.1 4	0.03
Second observation ADR, %, *n*	16.9 11	6.1 4	0.05

LCI: linked color imaging; WLI: white light; Ad: adenoma; SSA/P: sessile serrated adenoma and polyp; C: cecum; A: ascending colon; ASDR: adenoma and SSA/P detection rate; ADR: adenoma detection rate; SD: standard deviation.

**Table 4:** Overall results of the first and second observations.

	LCI 65 cases	WLI 65 cases	*p* value
Overall Ad and SSA/P numbers in the first and second observations	48	36	0.02
Overall Ad numbers in the first and second observations	43	30	0.02
Overall ASDR, %, *n*	43.1 28	35.4 23	0.36
Overall ADR, %, *n*	36.9 24	30.8 20	0.45

LCI: linked color imaging; WLI: white light; Ad: adenoma; SSA/P: sessile serrated adenoma and polyp; ASDR: adenoma and SSA/P detection rate; ADR: adenoma detection rate.

## Data Availability

The data used to support the findings of this study are available from the corresponding author upon request.
